# Development of a Phagocytosis-Dependent Gene Signature to Predict Prognosis and Response to Checkpoint Inhibition in Clear-Cell Renal Cell Carcinoma

**DOI:** 10.3389/fimmu.2022.853088

**Published:** 2022-05-16

**Authors:** Kunping Li, Yuqing Li, Yinfeng Lyu, Linyi Tan, Xinyi Zheng, Haowen Jiang, Hui Wen, Chenchen Feng

**Affiliations:** ^1^Department of Urology, Huashan Hospital, Fudan University, Shanghai, China; ^2^Department of Pharmacology, Huashan Hospital, Fudan University, Shanghai, China; ^3^Beijing Advanced Innovation Center for Food Nutrition and Human Health, Beijing Technology and Business University (BTBU), Beijing, China

**Keywords:** clear-cell renal cell carcinoma, antibody-dependent cellular phagocytosis, immune checkpoint inhibition, biomarker, bioinformatics

## Abstract

**Aim:**

The action of immune checkpoint inhibition (ICI) largely depends on antibody-dependent cellular phagocytosis (ADCP). We thus aim to develop ADCP-based ccRCC risk stratification as both prognostic and therapeutic markers of ICI.

**Method:**

Genomic data from multiple public datasets (TCGA, etc.) were integrated. A cancer-intrinsic ADCP gene set for ccRCC tailored from a recent report was constructed based on the association with prognosis, immune infiltrates, and response to ICI. Therapeutic potential was profiled using genome-drug sensitivity datasets.

**Results:**

ADCP genes were selected from a recent CRISPR/Cas9 screen report. Following a four-module panel based on clinical traits, we generated a six-gene signature (ARPC3, PHF19, FKBP11, MS4A14, KDELR3, and CD1C), which showed a strong correlation with advanced grade and stage and worsened prognosis, with a nomogram showing predictive efficacies of 0.911, 0.845, and 0.867 (AUC) at 1, 3, and 5 years, respectively. Signatures were further dichotomized, and groups with a higher risk score showed a positive correlation with tumor mutation burden, higher expressions of inhibitory checkpoint molecules, and increased antitumor immune infiltrates and were enriched for antitumor immune pathways. The high risk-score group showed better response to ICI and could benefit from TKIs of axitinib, tivozanib, or sorafenib, preferentially in combination, whereas sunitinib and pazopanib would better fit the low risk-score group.

**Conclusion:**

Here we showed a six-gene ADCP signature that correlated with prognosis and immune modulation in ccRCC. The signature-based risk stratification was associated with response to both ICI and tyrosine kinase inhibition in ccRCC.

## Highlights

* Antibody-dependent cellular phagocytosis (ADCP) is critical for action of immunotherapy and has yet been reported in kidney cancer.
* We for the first time developed an ADCP-based gene signature encompassing ARPC3, PHF19, FKBP11, MS4A14, KDELR3, and CD1C.
* The signature showed a strong correlation with prognosis and response to immunotherapy and may predict response to tyrosine kinase inhibitors.
* The signature holds promise as both a therapeutic and prognostic marker in kidney cancer.

## Introduction

Clear-cell renal cell carcinoma (ccRCC) is the most common subtype of renal cancer and is curable at the localized stage. Metastatic ccRCC consists of ~30% of cases and faces challenges in definitive treatment. Given the immunogenic nature of ccRCC, immunotherapy has re-immerged after initial attempts using interleukins and tumor-necrosis factor (TNF) (1, 2). The current frontline treatment for metastatic disease mandates the inclusion of immune checkpoint inhibition (ICI) that targets inhibitory immune checkpoints including PD-1, PD-L1, and CTLA-4 (3). Recent trials have demonstrated overwhelming advantages of the combination of ICI and tyrosine kinase inhibitors (TKIs), leaving monotherapy of TKI solely indicated for patients with favorable International Metastatic RCC Database Consortium (IMDC) risk (4).

However, the IMDC risk score is based on clinicopathological parameters that are dismally related to the pharmaceutical mechanisms of either TKI or ICI, let alone the synergy of combination (5). Besides the major anti-angiogenic effect and minor cancer-intrinsic inhibition effect of TKIs, the synergy is believed to result from increased immune infiltrates due to local inflammation by TKI and subsequent ICI antibodies kick in to facilitate the recognition of tumor cells by immune cells (6). Nonetheless, no consensus has been made on predictive marker(s) for ICI response even in a single type of cancer despite various attempts. PD-1/PD-L1 positivity, tumor mutation burden, and microsatellite instability (MSI) together with multiple experimental markers all showed efficacy in a certain context and can hardly be extrapolated across cancer types (7). Thus far, no genetic marker has been approved in the clinical setting for ccRCC.

Antibody-dependent cellular phagocytosis (ADCP) is the terminal step of ICI in which macrophages eliminate tumor cells upon previous activation of recognition (8). Albeit abundant in number, negative regulatory factors that dampen phagocytic activity remain the key obstacle hampering the full efficacy of ICI (9, 10). In ccRCC, the objective response rate of lenvatinib plus pembrolizumab reached 71%, among which 16.1% had complete response (11). While it is plausible to see a rapid change in the treatment paradigm in ccRCC, there are still ~30% of cases that fail to benefit from the combination. Whether ADCP plays a role in identifying potential beneficiaries from ICI and TKI in ccRCC remains unreported, and we thus aim to evaluate both prognostic and predictive merits with an *in silico* approach.

## Materials and Methods

### Data Acquirement and Processing

Processed and standardized RNA-seq data and clinical data of The Cancer Genome Atlas of Kidney Renal Clear Cell Carcinoma (TCGA-KIRC) cohort were downloaded from UCSC Xena (https://xena.ucsc.edu/). The E-MTAB-1980 dataset was acquired from the ArrayExpress database (https://www.ebi.ac.uk/arrayexpress/). Patients without complete prognostic data were eliminated. Then, patients with overall survival (OS) for more than 30 days were included in the subsequent analysis. The gene hits, identified with the CRISPR/Cas9 screen according to the 5% false discovery rate (FDR) or 95% credible interval, were collected from the previous research (8). These genes were defined as antibody-dependent cellular phagocytosis (ADCP)-related genes. Details on study designs have been published in a previous research (8).

### Co-Expression Module Construction

Weighted gene co-expression network analysis (WGCNA) is a system biology method used to describe the correlation patterns between different genes based on expression data. ADCP-related genes in TCGA-KIRC cohort were used to perform WGCNA analysis by the WGCNA R software package (12). GoodSamplesGenes function was used to eliminate outlier samples and genes. The appropriate soft power value was determined according to scale independence (more than 0.8). According to topological overlap matrix (TOM)-based dissimilarities, ADCP-related genes with similar expression profiles were classified into the same gene modules. The minimum number of genes was set as 30. The correlation between the module eigengene and the phenotype was evaluated by the Spearman correlation test. To functionally annotate different gene modules, the Metascape database (http://metascape.org) was utilized to annotate and visualize Gene Ontology (GO) and Kyoto Encyclopedia of Genes and Genomes (KEGG) enrichment analysis.

### Gene Signature Construction

The gene module with the highest correlation with clinical phenotypes was enrolled in a subsequent construction of gene signature. Univariate COX regression was performed on the training set, consisting of 97 ADCP-related genes and 513 ccRCC patients from TCGA-KIRC cohort, to select prognostic genes, and a cutoff of p-value was 0.01. Least absolute shrinkage and selection operator (LASSO) regression analysis is a common shrinkage method which can screen appropriate variables from multicollinear and high-dimensional data. In our study, LASSO regression was implemented on prognostic genes from the previous step using the “glmnet” R software package to screen the most valuable prognostic candidates. Next, stepwise regression was used to further shrink variables and select the best model based on the minimum Akaike information criterion (AIC) value principle by the “My.stepwise” R software package. Finally, the gene signature was constructed by multivariate COX regression. Based on the gene signature, the risk score of each patient was calculated by the formula below:


$$Riskscore\;=\sum_{i}Coefficient\;\left(i\right)*Expression\;of\;gene\;\left(i\right)$$

*Coefficient* (*i*) is the regression coefficient of *gene* (*i*) in the multivariate COX regression model; *Expression of gene* (*i*) is the expression value of (*i*). Patients were dichotomized into high- and low-risk groups based on risk scores. The survival analysis of different risk groups and genes in signature was performed by “survival” R software packages. Additionally, the “survminer” R software package was used to calculate the optimal cutoff value of gene expression. The “survivalROC” R software package was applied to plot receiver operating characteristic (ROC) curves to estimate the predictive accuracy of the gene signature. The E-MTAB-1980 cohort was utilized to validate the above results.

### Nomogram Construction

A nomogram was constructed based on risk score and clinical phenotypes by multivariate COX regression through “rms” and “survminer” R software packages. All-subset regression was performed to find the optimal model by the “leaps” R software package. The consistency between predicted and actual survival outcomes was evaluated according to calibration curves. Time-dependent ROC curves were plotted to estimate the predictive accuracy of the nomogram.

### Somatic Variant Analysis

Genetic mutation information was downloaded from TCGA database. The “maftools” package was used to calculate tumor mutation burden (TMB), visualize, and compare the frequency of genetic mutation between high- and low-risk groups. Copy number alteration (CNA) data of TCGA-KIRC were downloaded from the cBioPortal database (https://www.cbioportal.org/). The CNA burden was defined as the total number of genes with copy number gain or loss (13, 14).

### Identification of Differentially Expressed Genes and Functional Enrichment Analysis

The differentially expressed genes (DEGs) between high- and low-risk groups were identified by the limma R package (15). GO and KEGG enrichment analyses of upregulated genes in the high-risk group were implemented and visualized by the “clusterProfiler” R package (16). Gene set enrichment analysis (GSEA) was performed to analyze significantly enriched pathways in the high-risk group by “GSEA” software. In GSEA, significantly enriched pathways met the following criteria: normalized enrichment score >1; nominal p-value < 0.05; and FDR q-value < 0.25. The gene set of “c5.go.bp.v7.4.symbols.gmt” was selected to perform GSEA.

### Immune Cell Infiltrate Analysis

ESTIMATE, CIBERSORT, and single-sample gene set enrichment analysis (ssGSEA) algorithms were applied to calculate the abundance of immune cell infiltrations between high- and low-risk groups (17–19).

### Correlation of the Gene Signature With Immunotherapy

Immunophenoscore (IPS) has been verified as a signature to predict the response to immunotherapy (20). The IPS score was calculated based on the expression of the representative genes or gene sets: immunomodulators, MHC molecules, effector cells, and suppressor cells. The IPS score of TCGA-KIRC was downloaded from The Cancer Immunome Atlas (TCIA) database (https://tcia.at/home). RNA-seq profiles and clinical data of anti-PD-1 antibody nivolumab in ccRCC were derived from three clinical trials: CheckMate 009 (CM-009; NCT01358721), CheckMate 010 (CM-010; NCT01354431), and CheckMate 025 (CM-025; NCT01668784) (21–23). The normalized and integrated data were downloaded from the supplementary data of the previous research (24). Risk scores for the above patients were calculated based on the gene signature.

### Prediction of Chemotherapeutic Effect and Exploring Potential Chemotherapy Drugs in ccRCC

For personalized treatment, the sensitivity of chemotherapy was predicted based on the Genomics of Drug Sensitivity in Cancer (GDSC, https://www.cancerrxgene.org/) database. The “pRRophetic” R package (25) was applied to estimate the half-maximal inhibitory concentration (IC50) of some common chemotherapy drugs in ccRCC. The DEGs between two risk groups were uploaded into the connectivity map (cMAP, https://portals.broadinstitute.org/cmap/) database (26) to explore potential chemotherapy drugs. The enrichment score varied from -1 to 1. The cutoff of the p-value was 0.05.

### Sample Collection and Immunohistochemistry

A standard immunohistochemistry (IHC) protocol was followed. In brief, sections were sliced consecutively at 4 μm and were then deparaffinized followed by gradient rehydration in ethanol and subsequently rinsed. Samples were then mounted on polylysine-coated glass. A pilot staining was conducted using multiple antibodies. Multiple primary antibodies were tested, and antibodies against FKBP11 (Sigma-Aldrich, St. Louis, MO, USA, Cat. HPA041709; dilution 1:500), ARPC3 (Sigma-Aldrich, Cat. HPA006550; dilution 1:500), KDELR3 (Sigma-Aldrich, Cat. HPA043477; dilution 1:500), and MS4A14 (Abcam, Cat. ab182151) were adopted for the final evaluation. Assessment of positivity was referenced from The Human Protein Atlas (27), as none of the factors were reported before in ccRCC using IHC staining. According to the intensity and extensity of staining, we designated a three-tiered scoring system for FKBP11 and ARPC3 and a dichotomized scoring system for KDELR3 and MS4A14. However, CD1C and PHF19 were not IHC-detectable in ccRCC despite multiple trials with different antibodies, which was also supported in The Human Protein Atlas (27). The sections treated with PBS in lieu of the primary antibody were chosen as negative controls, and tumor-infiltrating lymphocytes (TILs) were stained and evaluated using the H-score, as previously reported (28). All patients signed an informed consent, and the study was approved by the Huashan Institutional Review Board (HIRB-2022-204).

### Statistical Analysis

The Kaplan–Meier log-rank test was applied to perform survival analysis. The Wilcoxon test was used to evaluate continuous-variable data. The chi-square test and Fisher’s exact test were performed to analyze differences between categorical variables. The Spearman correlation analysis was used to calculate the correlation coefficient. The IHC data were regarded as non-parametric, and comparisons for medians were analyzed using Mann–Whitney’s U-test or Kruskal–Wallis test with *post-hoc* Dunn’s test. The p value of <.05 was accepted as statistically significant. The visualization of results was performed by “ggplot2,” “ggpubr,” and “ggstatsplot” R software packages. Statistical analysis and visualization were implemented by R software (version 4.1.1) and GraphPad software.

## Results

### Data Acquirement

**Figure 1:**
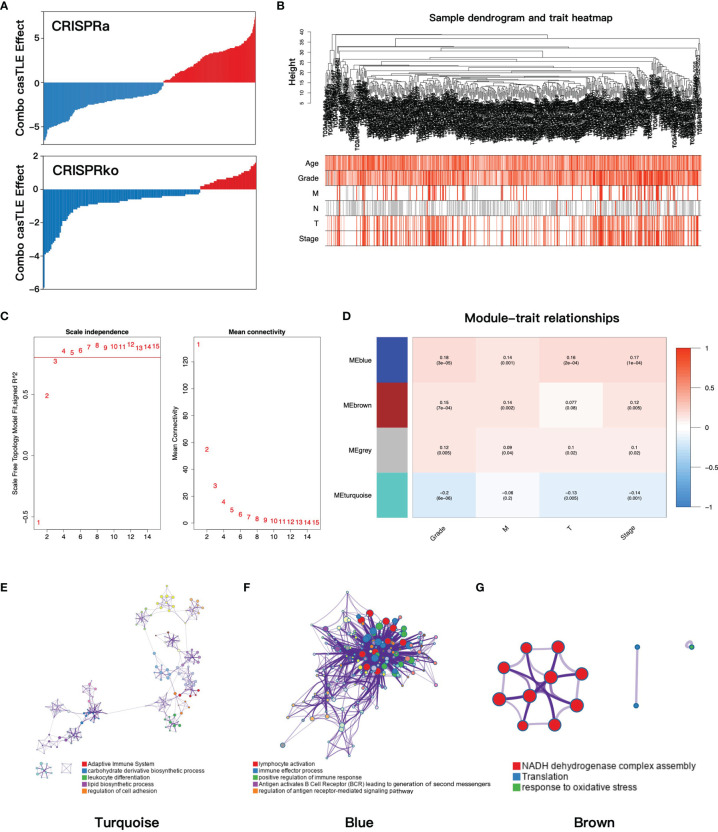
**(A)** The casTLE score of ADCP-related genes in the CRISPR/Cas9 screen. **(B)** Clustering dendrogram and clinical traits heatmap of ccRCC patients based on ADCP-related genes. The shade of color in the heatmap increased with age, Fuhrman grade, pathology T stage, and pathology stage. Red, white, and gray are on behalf of metastasis, no metastasis, and data missing in pathology M and N stage, respectively. **(C)** The relationship between the soft threshold and the index of scale-free topologies and mean connectivity. **(D)** The heatmap of the correlation of gene modules and clinical traits. In each grid, the top number was the coefficient between gene modules and clinical traits, and the bottom was the p-value. **(E–G)** The functional clustering network of turquoise, blue, and brown modules. casTLE, cas9 high-throughput maximum likelihood estimator; ADCP, antibody-dependent cellular phagocytosis; ccRCC, clear cell renal cell carcinoma.

The training set consisted of 513 ccRCC patients and 72 normal samples from TCGA-KIRC cohort. The testing set consisted of 100 ccRCC patients from the E-MTAB-1980 cohort. The detailed clinical information of training and testing cohorts is shown in **Table 1**. A total of 543 ADCP-related genes were extracted from a previous research (8), of which 511 were found in TCGA cohort (**Figure 1A**). In the CRISPR activation (CRISPRa) screen, genes with the combo casTLE effect less than 0 were considered as anti-phagocytic genes, whereas those with the combo casTLE effect more than 0 were pro-phagocytic genes. In the CRISPR knockout (CRISPRko) screen, the definition was the opposite. casTLE, which stands for cas9 High Throughput maximum Likelihood Estimator, was utilized to evaluate the gene effect sizes comparing negative controls in the CRISPR/Cas9 screen (29). Details on study designs and process of genes screening could refer to the previous research (8).

**Table 1:** Clinical and pathologic features of eligible ccRCC patients from TCGA-KIRC and E-MTAB-1980.

Features	TCGA-KIRC (training set)	E-MTAB-1980 (testing set)
**Total**	513	100
**Age, n (%)**		
**≤65 years**	173 (33.7)	56 (56)
**>65 years**	340 (66.3)	44 (44)
**Sex, n (%)**		
**Female**	176 (34.3)	24 (24)
**Men**	337 (65.7)	76 (76)
**Fuhrman grade, n (%)**		
**GX**	8 (1.6)	2 (2)
**G1**	12 (2.3)	13 (13)
**G2**	218 (42.5)	59 (59)
**G3**	202 (39.4)	21 (21)
**G4**	73 (14.2)	5 (5)
**AJCC T stage, n (%)**		
**T1**	261 (50.9)	67 (67)
**T2**	68 (13.3)	11 (11)
**T3**	173 (33.7)	21 (21)
**T4**	11 (2.1)	1 (1)
**AJCC N stage, n (%)**		
**NX**	268 (52.3)	0 (0)
**N0**	229 (44.6)	93 (93)
**N1**	16 (3.1)	7 (7)
**AJCC M stage, n (%)**		
**MX**	28 (5.5)	0 (0)
**M0**	407 (79.3)	88 (88)
**M1**	78 (15.2)	12 (12)

ccRCC, clear cell renal cell carcinoma; KIRC, kidney renal clear cell carcinoma; AJCC, American Joint Committee on Cancer.

### Construction of Co-Expression Modules and Identification of the Key Module

To find the gene modules with the highest correlation with clinical phenotypes, we implemented WGCNA analysis on TCGA-KIRC cohort. After deleting outliers and unqualified samples, 507 patients and 511 ADCP-related genes were enrolled into the subsequent analysis. The sample dendrogram and heatmap of clinical phenotypes are presented in **Figure 1B**. In order to construct the gene co-expression network, the optimal soft-thresholding value was set as 4 (scale-free R2 = 0.85, mean connectivity = 15.99, **Figure 1C**) and cut height was set as 0.5, after which the four-gene modules were identified (turquoise 369 genes, blue 97 genes, gray 6 genes, and brown 39 genes). Next, the correlations between gene modules and clinical traits were evaluated to find the key module, and the result showed that the blue module was most correlated with clinical features (**Figure 1D**). Then, we performed functional enrichment analysis to evaluate the biological function of each gene module based on the Metascape database. For the turquoise module, the top enriched terms were adaptive immune system, carbohydrate derivative biosynthetic process, and leukocyte differentiation (**Figure 1E**). For the blue module, lymphocyte activation, immune effector process, and positive regulation of immune response were significantly enriched (**Figure 1F**), which indicated that the blue module played important roles in immune regulation. Finally, the brown module was enriched in NADH dehydrogenase complex assembly, translation, and response to oxidative (**Figure 1G**).

### Construction of the Gene Signature

**Figure 2:**
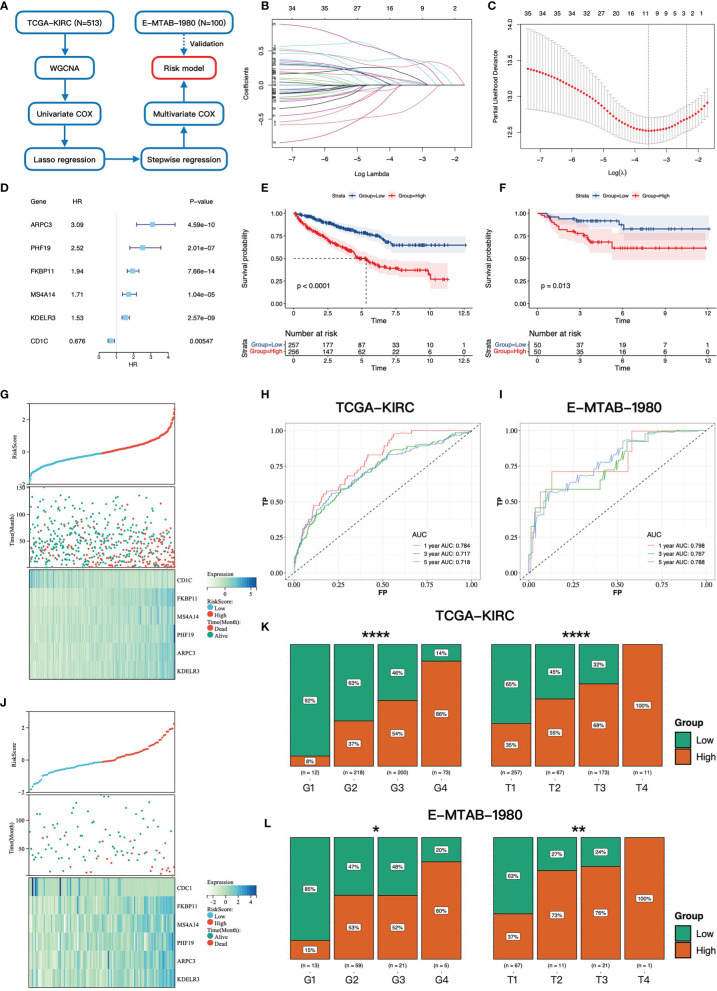
Construction and validation of the gene signature. **(A)** The workflow of the constructing gene signature. **(B)** The coefficients of LASSO regression in the 10-fold cross validation based on 38 candidate genes. **(C)** Ten-fold cross-validation for tuning parameter selection. The dotted vertical lines are drawn at the optimal values by the minimum criteria and one-standard error criterion. **(D)** The forest plot of six hub genes based on univariate COX regression of overall survival in TCGA-KIRC cohort. **(E, F)** Kaplan–Meier survival curves of overall survival for patients in high- and low-risk groups in TCGA-KIRC cohort and E-MTAB-1980 cohort. **(G, J)** The distributions of the risk score and vital status in TCGA-KIRC cohort and E-MTAB-1980 cohort. **(H, I)** The time-dependent ROC curves at 1, 3, and 5 years based on the gene signature in TCGA-KIRC cohort and E-MTAB-1980 cohort. The proportion of high- and low-risk groups in different Fuhrman grades and pathology T stages in TCGA-KIRC cohort **(K)** and E-MTAB-1980 cohort **(L)** * 0.01 < p < 0.05; ** 0.001 < p < 0.01; ****p < 0.0001.

To explore the prognostic value of ADCP-related genes, we constructed a gene signature based on TCGA-KIRC cohort. The workflow of the constructing gene signature is shown in **Figure 2A**. The genes in the blue module, which was most correlated with clinical phenotypes, were selected as candidate genes of the constructing gene signature. Taking advantage of univariate COX regression, we firstly screened 38 prognostic genes (p-value < 0.01, **Supplementary Data Table S1**) from the blue module. To avoid multicollinearity and overfitting, we then applied LASSO regression on these prognostic genes and recognized nine genes more related to prognosis in ccRCC (**Figures 2B, C**). Next, to further screen variates, stepwise regression was performed to identify the optimal model based on the principle of minimum AIC. Finally, FKBP11 (FKBP Prolyl Isomerase 11), CD1C (CD1c Molecule), PHF19 (PHD Finger Protein 19), ARPC3 (Actin Related Protein 2/3 Complex Subunit 3), MS4A14 (Membrane Spanning 4-Domains A14), and KDELR3 (KDEL Endoplasmic Reticulum Protein Retention Receptor 3) were incorporated into the multivariate COX regression model and risk score = (0.261 * FKBP11 expression) + (-0.627 * CD1C expression) + (0.667 * PHF19 expression) + (0.418 * ARPC3 expression) + (0.298 * MS4A14 expression) + (0.155 * KDELR3 expression) (**Figure 2D**). The risk score of each patient was calculated by this model, and each patient was divided into high- and low-risk groups according to the median of risk score.

### High-Risk Group Correlated With Worse Prognosis, Higher Fuhrman Grade, and Advanced Pathologic Stage

**Figure 3:**
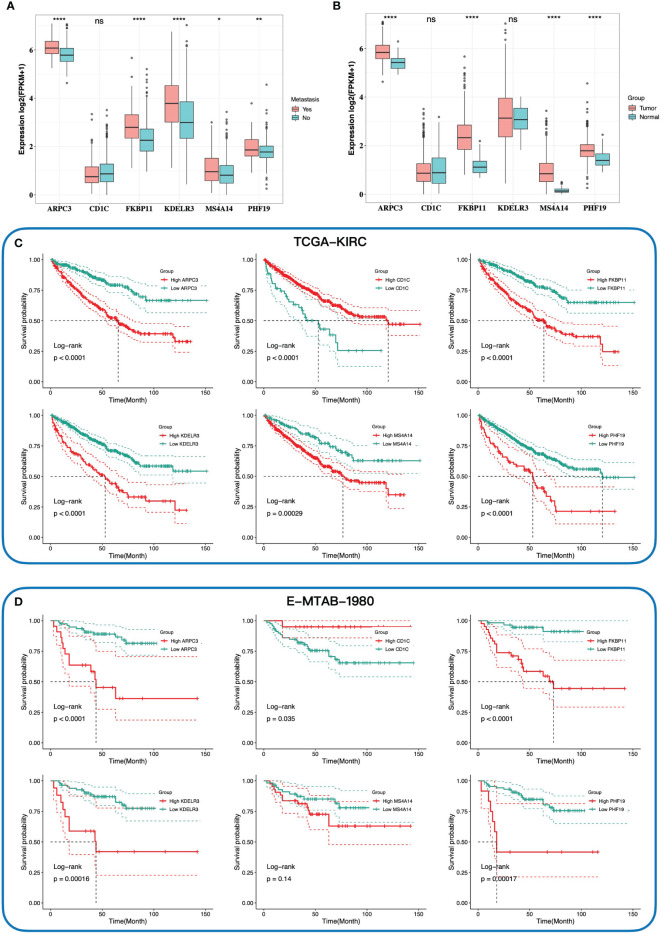
The relationship between expression of six key genes and tumor, metastasis, and prognosis. The comparison expression of six hub genes in patients with and without distant metastasis **(A)**, and tumor tissues and normal tissues **(B)**. Kaplan–Meier survival curves of the overall survival of six hub genes in TCGA-KIRC cohort **(C)** and E-MTAB-1980 cohort **(D)** according to the optimal cutoff value of gene expression. * 0.01 < p < 0.05; ** 0.001 < p < 0.01; ****p < 0.0001; ns no significance.

We then investigated the association between the gene signature and clinical phenotypes. In the training cohort, most of the dead cases were concentrated in the high-risk group (**Figure 2G**). Except for CD1C, other genes were upregulated in the high-risk group according to the heatmap (**Figure 2G**). The result of survival analysis demonstrated that the high-risk group had a worse prognosis than the low-risk group (p-value < 0.0001, **Figure 2E**). Additionally, the AUC (area under the curve) values of the gene signature at 1, 3, and 5 years were 0.784, 0.717, and 0.718, respectively (**Figure 2H**). Furthermore, with the elevation of grades and stages, the proportions of the high-risk group were significantly increased accordingly (**Figure 2K**). In the testing cohort, the above conclusions were consistent with the training cohort. More dead cases distributed in the high-risk group (**Figure 2J**). CD1C was overexpressed in the low-risk group, whereas the expressions of the others were upregulated in the high-risk group. The prognosis in the high-risk group was worse likewise (p-value = 0.013, **Figure 2F**). A higher predictive accuracy was presented in the E-MTAB-1980 cohort, and the AUC values at 1, 3, and 5 years were 0.798, 0.767, and 0.788 (**Figure 2I**). The distribution of the high- and low-risk groups in different stages and grades was consistent with the training cohort (**Figure 2L**). Finally, we investigated the relationship between six key genes and prognosis and clinical phenotypes. FKBP11, PHF19, ARPC3, MS4A14, and KDELR3 were significantly upregulated in M1 cases (**Figure 3A**). FKBP11, PHF19, ARPC3, and MS4A14 were overexpressed in tumor tissues (**Figure 3B**). Moreover, based on the optimal cutoff value of gene expression, higher expressions of FKBP11, PHF19, ARPC3, MS4A14, and KDELR3 had a worse prognosis in the training and testing cohorts, while patients with higher-level CD1C had a more prolonged OS in both cohorts (**Figures 3C, D**). The above conclusions indicate that the gene signature has a good performance in predicting prognosis and a robust correlation with clinical phenotypes. In addition, the gene signature is stable and verifiable.

### The Gene Signature for Nomogram Construction

**Figure 4:**
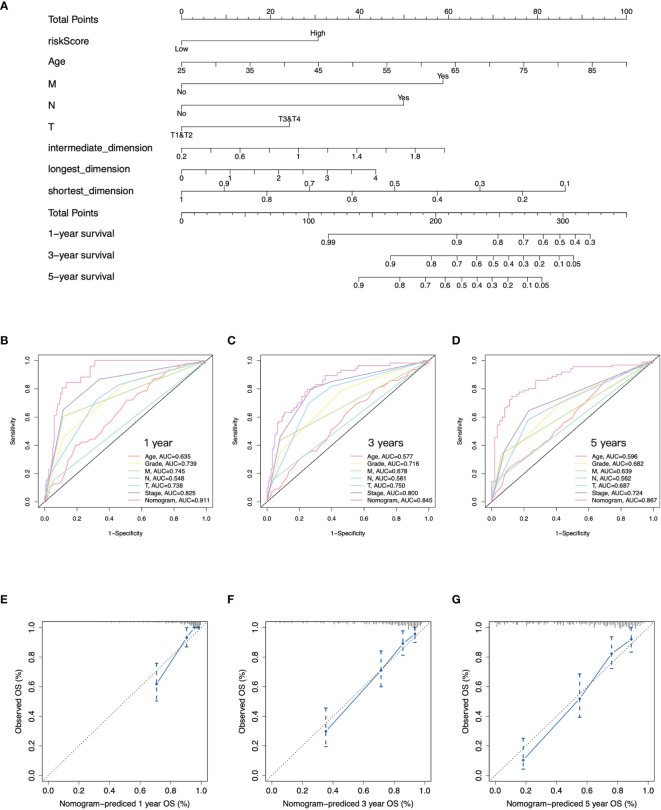
Construction of nomogram. **(A)** Nomogram to predict overall survival at 1, 3, and 5 years based on the gene signature, age, pathology T, N, and M stages, and tumor size. **(B–D)** Time-dependent ROC curves at 1, 3, and 5 years of the nomogram and clinical features of ccRCC. **(E–G)** Calibration curves of nomogram for predicting overall survival at 1, 3, and 5 years.

To quantitatively predict the prognosis of ccRCC, we integrated the gene signature and clinical phenotypes of patients from TCGA-KIRC cohort to construct a predictive nomogram. According to the median of risk score, patients were divided into high- and low-risk groups. Patients then were further subdivided into different subgroups: distant metastasis yes and no, lymph node metastasis yes and no, T1/T2, and T3/T4. The all-subset regression method was performed, and the model with maximal adjust R square was selected. Finally, risk score, age, distant metastasis, lymph node metastasis, pathology T stage, and shortest, intermediate, and longest-dimension of primary tumor were incorporated into multivariate COX regression analysis to construct the nomogram (**Figure 4A**). The overall survival at 1, 3, and 5 years for each patient could be predicted based on the total points from the nomogram. Furthermore, the time-dependent ROC curves presented that the nomogram had the highest AUC values at 1, 3, and 5 years of 0.911, 0.845, and 0.867, respectively, compared to other clinical features of ccRCC (**Figures 4B–D**). There was a good consistency between the actual observed OS and predictive OS by nomogram according to the calibration curves at 1, 3, and 5 years (**Figures 4E–G**). Taken together, the nomogram had a good performance on predictive accuracy in TCGA-KIRC cohort; however, there was no independent external cohort to validate it due to lack of tumor size data.

### Somatic Variant Analysis Between High- and Low-Risk Groups

**Figure 5:**
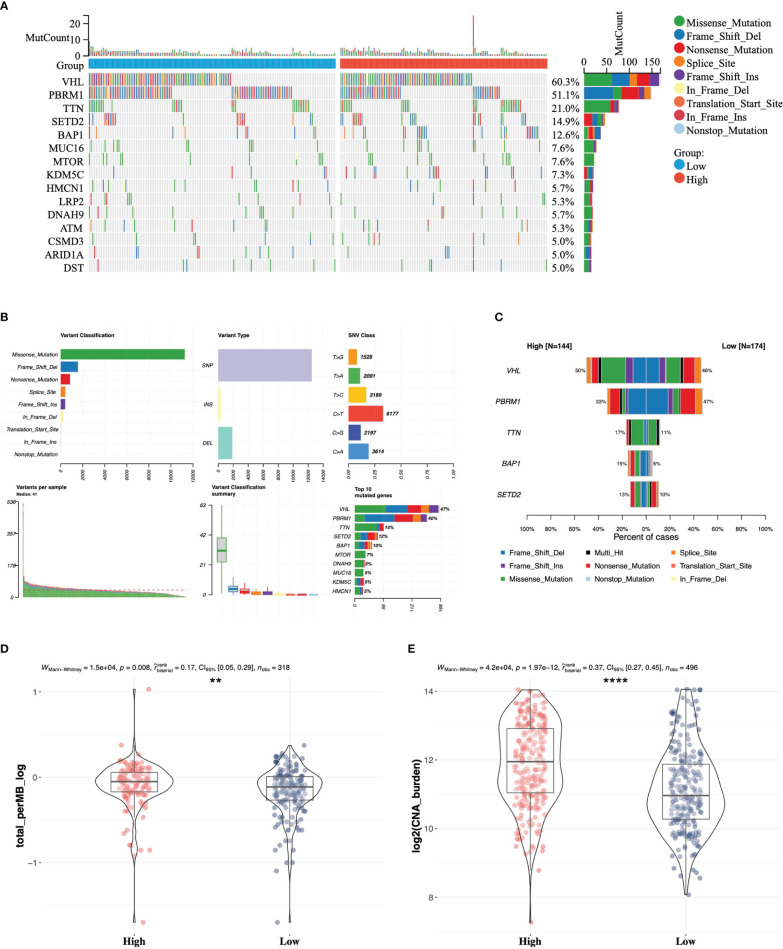
Somatic variant analysis between high- and low-risk groups in TCGA. **(A)** Top 15 mutated genes in high- and low-risk groups. **(B)** Landscape of somatic mutations in TCGA-KIRC cohort. **(C)** Comparison of top five mutated genes in high- and low-risk groups. Differences in tumor mutation burden **(D)** and copy number alteration burden **(E)** between the high- and low-risk groups ** 0.001 < p < 0.01; ****p < 0.0001.

As somatic gene mutation could act as trunk events promoting tumorigenesis, especially in renal cell carcinoma, we figured out top 15 mutated genes in high- and low-risk groups separately (**Figure 5A**) and summarized variant classification displaying the number of variants in each sample as a stacked bar plot and variant types as a boxplot (**Figure 5B**). We identified the top five differentially mutated genes (**Figure 5C**) including VHL, PBRM1, TTN, BAP1, and SETD2 in high- and low-risk groups. The high-risk group with worse prognosis possessed a higher frequency of BAP1 mutation, and a previous study has proved that BAP1 loss is associated with high tumor grade (30). What is more, the frequency of PRBM1 mutation was higher in the low-risk group. A previous study has shown that PRBM1 is associated with angiogenesis and patients with PRBM1 mutation are more likely to benefit from antiangiogenics than from immunotherapy (31). We also compared tumor mutation burden (TMB) (**Figure 5D**) and copy number alteration (CNA) burden (**Figure 5E**), which have been considered as response biomarkers for immune checkpoint blockade in solid tumors such as melanoma, non-small cell lung cancer, and urothelial carcinoma. In our study, it was found that patients in the high-risk group had significantly higher TMB and CNA burden. According to the evidence that tumors with higher TMB implied better ICI efficacy (32), we speculated that the high-risk group with higher genomic alteration burden possessed a better response to anti-PD-1 or anti-PD-L1 antibody treatment in ccRCC.

### Functional Enrichment Analysis in High- and Low-Risk Groups

**Figure 6:**
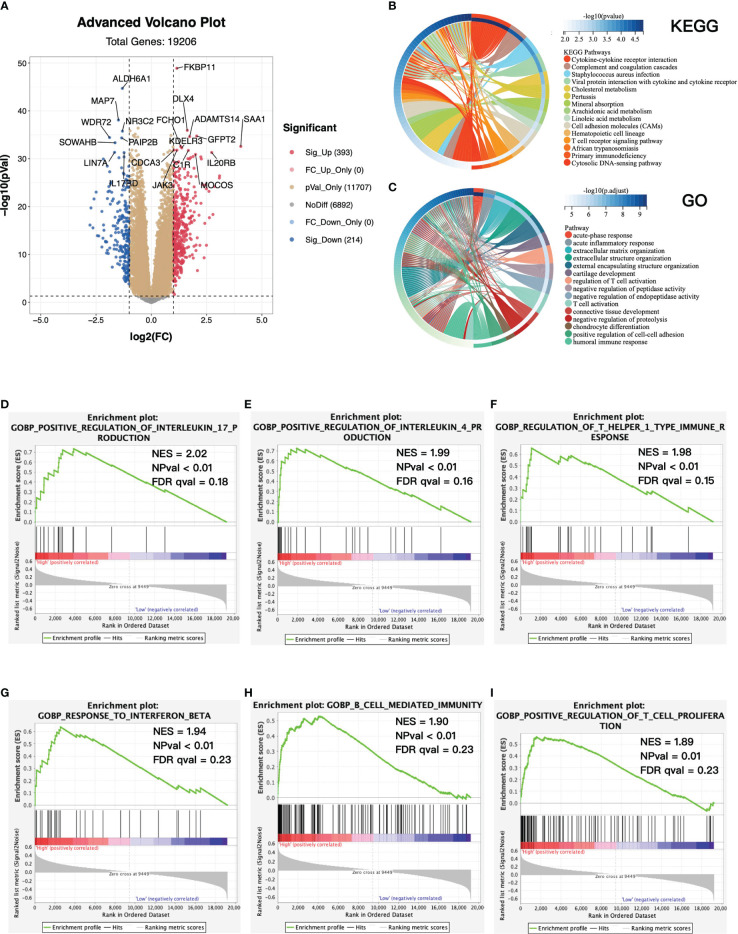
Functional enrichment between high- and low-risk groups. **(A)** Volcano plot of differentially expressed genes (DEGs) between high- and low-risk groups. Log2(fold change) > 1, adjusted p-value < 0.05. KEGG **(B)** and GO **(C)** enrichment analysis base on upregulated DEGs. Top 15 enriched pathways were presented. **(D–I)** GSEA between high- and low-risk groups. KEGG, Kyoto Encyclopedia of Genes and Genomes; GO, Gene Ontology; GSEA, gene set enrichment analysis; NES, normalized enrichment score; NPval, nominal p-value; FDR qval, FDR q-value.

To further estimate the biological difference in distinct risk groups, we screened out differentially expressed genes (DEGs) between the high- and low-risk groups. A total of 393 genes were significantly upregulated in the high-risk group, while 214 genes were downregulated (**Figure 6A**). KEGG and GO analyses were performed based on 393 overexpressed genes in the high-risk group (**Figures 6B, C**). We found that the cytokine–cytokine receptor interaction and T-cell receptor signaling pathway in KEGG, and acute inflammatory response, regulation of T-cell activation, and T-cell activation in GO were enriched in the high-risk group. Similarly, GSEA showed that the high-risk group was significantly associated with several antitumor immune pathways (**Figures 6D–I**), which contained positive regulation of interleukin 17 production, positive regulation of interleukin 4 production, regulation of T helper I type immune response, response to interferon β, B cell-mediated immunity, and positive regulation of T-cell proliferation. To sum up, we revealed the biological differences between high- and low-risk groups mainly associated with immune-related pathways, and the high-risk group was significantly associated with antitumor immune pathways.

### Immune Landscape of High- and Low-Risk Groups

**Figure 7:**
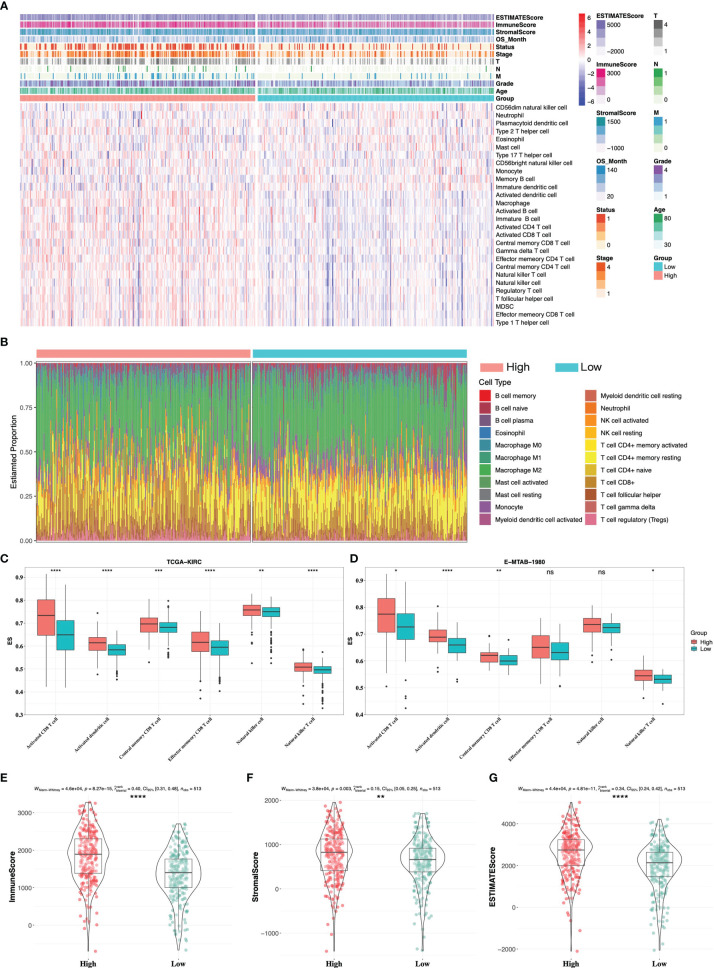
Immune infiltrate landscape of high- and low-risk groups. **(A)** The landscape of immune infiltrates and clinical phenotypes between high- and low-risk groups. **(B)** The relative proportion of 22 immune cells in high- and low-risk groups by CIBERSORT analysis. Antitumor-related immune cells between high- and low-risk groups in TCGA-KIRC cohort **(C)** and E-MTAB-1980 cohort **(D)**. **(E–G)** ESTIMATE analysis between high- and low-risk groups in TCGA-KIRC cohort. * 0.01 < p < 0.05; ** 0.001 < p < 0.01; *** 0.0001 < p < 0.001; ****p < 0.0001; ns no significance.

We then evaluated tumor immune cell infiltration in the tumor microenvironment (TME) between high- and low-risk groups. The landscape of immune infiltration and clinical parameters displayed higher grade, advanced TNM stages, and more decreased events in the high-risk group (**Figure 7A**). CIBERSORT analysis was also used to assess the proportion of 22 lymphocytes in the TME of ccRCC (**Figure 7B**), and the result showed that the TME of the high-risk group was composed of more T-cell CD4+ memory activated/resting, T-cell CD4+ naïve, T-cell CD8+, and less macrophage M2. Specifically, the abundance of antitumor immune cells, including activated CD8 T cell, activated dendritic cell, central memory CD8 T cell, effector memory CD8 T cell, natural killer cell, and natural killer T cell, was higher in the high-risk group in TCGA and E-MTAB-1980 cohorts (**Figures 7C, D**). Besides, by using the ESTIMATE algorithm on TCGA-KIRC cohort, patients in the high-risk group had higher immune score, stromal score, and ESTIMATE score (**Figures 7E–G**).

### High-Risk Group Was Associated With a Better Response to Immunotherapy

**Figure 8:**
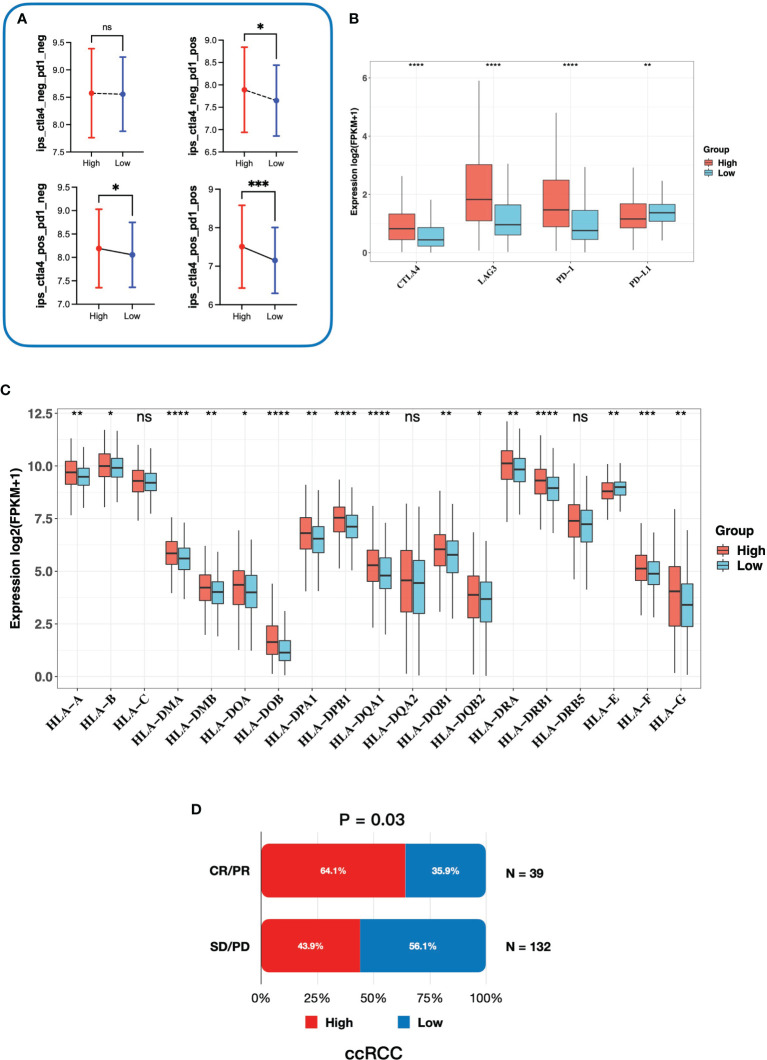
Correlation between the gene signature and response to immunotherapy. **(A)** The relationship between immunophenoscore and the gene signature. **(B)** Expression of inhibitory immune checkpoints in high- and low-risk groups. **(C)** Expression of the HLA gene family in high- and low-risk groups. **(D)** The association between risk groups and response to anti-PD-1 antibody treatment in the ccRCC cohort. CR, complete response; PR, partial response; SD, stable disease; PD, progressive disease. * 0.01 < p < 0.05; ** 0.001 < p < 0.01; *** 0.0001 < p < 0.001; ****p < 0.0001; ns no significance.

To investigate whether the gene signature was related to the response to immunotherapy, we firstly performed IPS analysis on TCGA-KIRC cohort. In our study, the IPS-PD1/PDL1/PDL2 blocker score (PD-1/PD-L1/PD-L2 positive and CTLA-4 negative), IPS–CTLA4 blocker score (PD-1/PD-L1/PD-L2 negative and CTLA-4 positive), and IPS–CTLA4 and PD1/PDL1/PDL2 blocker score (PD-1/PD-L1/PD-L2 positive and CTLA-4 positive) were significantly higher in the high-risk group (**Figure 8A**), which indicated that patients with a high risk score might benefit from immunotherapy. Next, we explored expressions of the common inhibitory immune checkpoints (ICPs) in the high- and low-risk groups. We found that there were more inhibitory immune checkpoints (CTLA4, LAG3, and PD-1) significantly upregulated in the high-risk group (**Figure 8B**). In addition, it has been proved that the loss of the HLA gene family is associated with resistance to immunotherapy (33, 34). In our study, most of HLA genes were overexpressed in the high-risk group (**Figure 8C**), which further proved that patients in the high-risk group might be suitable for immunotherapy than the low-risk group. Moreover, in order to further elaborate the relationship between gene signature and response to immunotherapy, we directly validated it in a ccRCC cohort treated with the anti-PD-1 antibody. The ccRCC cohort consisted of 39 CR/PR (complete response/partial response) patients and 132 SD/PD (stable disease/progressive disease) patients. As is shown in **Figure 8D**, in the CR/PR group, patients with a higher risk score occupied more proportion (64.1% vs. 35.9%, p-value = 0.03). Taken together, patients with a higher risk score might be the optimal beneficiaries for immunotherapy and we provided a novel and potential gene signature to predict response to immunotherapy.

### Functional Analysis of Six Key Genes

**Figure 9:**
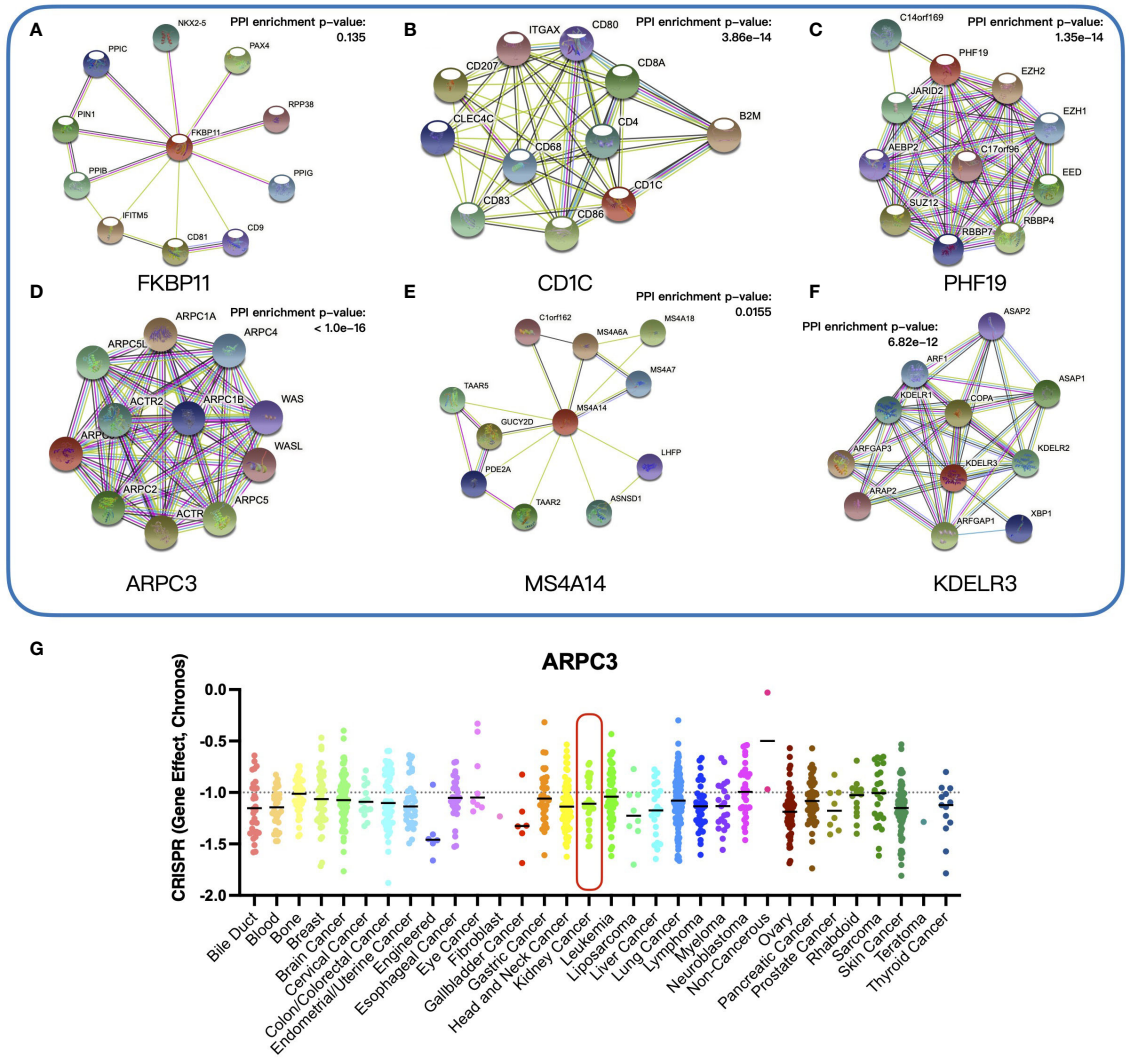
Functional analysis of six key genes. **(A–F)** Protein–protein interaction networks of six key genes. **(G)** The Chronos dependency scores of ARPC3 gene in 31 tumor types.

To investigate the function of six key genes, we constructed the protein–protein interaction (PPI) networks evaluated by the STRING database. Within the PPI network, each node represented all the proteins produced by a single, protein-coding gene locus and each edge represented protein–protein associations. FKBP11 was mainly enriched in the biological process of the GO term associated with myoblast fusion involved in skeletal muscle regeneration, but the PPI network was of no statistical significance (**Figure 9A**). CD1C was enriched in several immune-related biological processes, such as positive regulation of interleukin-2 production (**Figure 9B**). PHF19 was mainly correlated with histone methylation (**Figure 9C**). ARPC3 was primarily involved in biological processes associated with meiosis (**Figure 9D**). However, MS4A14 was not enriched in any biological process of the GO term (**Figure 9E**). KDELR3 was associated with protein intracellular transport (**Figure 9F**). The detailed biological processes of GO terms about these genes are shown in **Supplementary Data Table S2**. In addition, we investigated the genetic dependencies of six key genes in cancer cells evaluated by the Cancer Dependency Map (DepMap) database. The Chronos dependency score, based on data from a cell depletion assay, was utilized to describe the importance of the gene of interest on a given cell line. A lower Chronos score indicated more essential to tumor cell proliferation. A score of 0 indicated a gene not essential; correspondingly, -1 means comparable to the median of all pan-essential genes. The results showed that only the medians of the Chronos score of ARPC3 were less than -1 in most tumor cells, including kidney cancer cells (**Figure 9G**), which suggested that ARPC3 was more essential to tumor cell proliferation.

### Prediction for Response to Chemotherapy Drugs and Investigation of Potential Drugs Based on the Gene Signature

**Figure 10:**
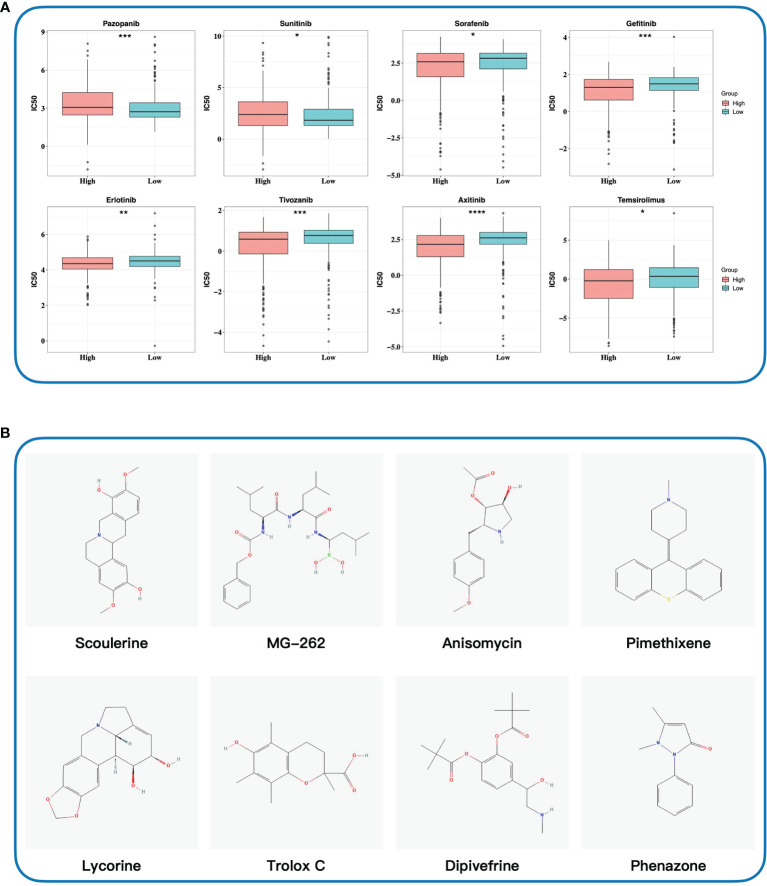
Exploring potential drugs. **(A)** Predicting half-maximal inhibitory concentration values of eight common chemotherapy drugs based on the GDSC database. **(B)** Identifying potential small-molecule drugs based on DEGs by the cMAP database. The 2D structures of top 10 small-molecule drugs were presented. IC50, half-maximal inhibitory concentration; GDSC database, Genomics of Drug Sensitivity in Cancer database; cMAP database, the connectivity map database. * 0.01 < p < 0.05; ** 0.001 < p < 0.01; *** 0.0001 < p < 0.001; ****p < 0.0001.

To select chemotherapy drugs suitable for patients in the high- and low-risk groups respectively, we predicted the IC50 values of common chemotherapy drugs in TCGA-KIRC based on the GDSC database. The results demonstrated that the IC50 values of pazopanib and sunitinib were lower in the low-risk group, whereas sorafenib, gefitinib, erlotinib, tivozanib, axitinib, and temsirolimus were lower in the high-risk group (**Figure 10A**). Based on the results, personalized treatments could be provided for each patient according to the risk score. Meanwhile, we utilized the cMAP database to explore small-molecule drugs which might be effective for ccRCC. Twelve small-molecule drugs were identified (p-value < 0.05, **Supplementary Data Table S3**) based on the 249 upregulated genes and 110 downregulated genes in the high-risk group. The 2D structures of the top 10 small-molecule drugs are presented on **Figure 10B**. Taken together, our gene signature may contribute to personalized treatment and the development of new drugs in ccRCC.

### Validated Prognostic Markers and Correlation With TILs

**Figure 11:**
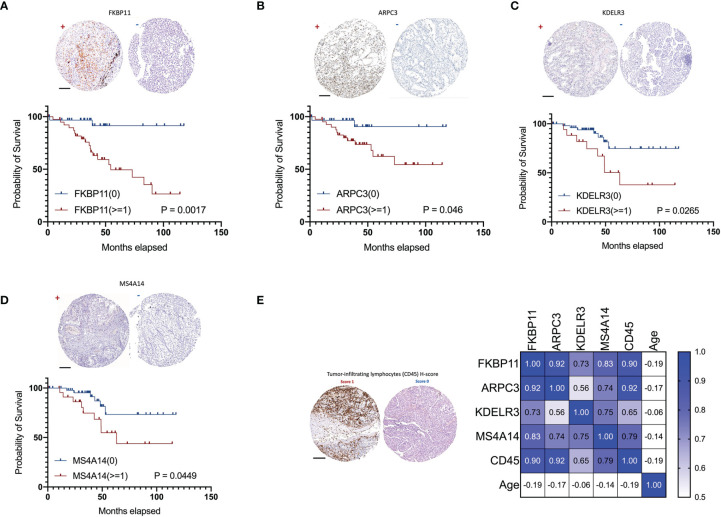
Validation of ADCP-related genes in 72 in-house ccRCC samples. Shown were representative IHC images (“+” for score >=1 and “-” for score 0) and impact on overall survival of **(A)** FKBP11, **(B)** ARPC3, **(C)** KDELR3, and **(D)** MS4A14. **(E)** Representative H-score 1 and 0 for TIL abundance and heatmap for its correlations with ADCP-related genes and age (Spearman’s r). (Scale bar = 200 µm).

Due to the availability and performance of IHC antibodies in the pilot assays, only FKBP11, ARPC3, KDELR3, and MS4A14 were validated for prognostics using our in-house FFPE samples with IHC staining (**Figures 11A–D**). A retrospective consecutive cohort comprising 72 samples from patients who underwent radical or partial nephrectomy from January 2009 to December 2011 was assembled for validation. We chose the patients from the last decade simply due to the relative latent disease course of ccRCC, and survival events were not mature in a more recent cohort. Demographic characteristics are shown in **Table 2**. Expressions of all four factors were associated with worsened overall survival and advanced tumor grade and stage, respectively (**Figures 11A–D**, **Table 2**). Of note, primary tumors in patients with metastasis (M1) also showed higher expressions of the four (**Table 2**). There was no gender disparity or age preference (**Table 2** and **Figure 11E**). All four factors showed a moderate to strong correlation with TIL abundancy (**Figure 11E**).

**Table 2:** Association of with clinicopathological parameters of ccRCC patients in the IHC validation cohort.

Parameter	Breakdown	N	FKBP11		p	ARPC3		p	KDELR3		p	MS4A14		p
			Median	SEM		Median	SEM		Median	SEM		Median	SEM	
**T**	T1	41	0	0.07	<0.0001	0	0.08	<0.0001	0	0.00	<0.0001	0	0.00	<0.0001
	T2	14	2	0.07		2	0.00		0	0.14		1	0.11	
	T3	17	3	0.11		2	0.08		1	0.10		1	0.08	
**N**	N0	65	1	0.14	0.0003	1	0.12	0.0184	0	0.05	0.0014	0	0.06	0.0076
	N1	7	3	0.30		2	0.18		1	0.14		1	0.14	
**M**	M0	67	1	0.14	0.001	1	0.11	0.0102	0	0.05	0.0011	0	0.06	0.0047
	M1	5	3	0.20		2	0.00		1	0.00		1	0.00	
**Gender**	Male	52	1	0.15	0.4763	1	0.12	0.0895	0	0.06	0.5576	0	0.07	>0.9999
	Female	20	0	0.33		0	0.22		0	0.11		0	0.11	
**Grade**	I	7	0	0.00	0.0022	0	0.00	0.0049	0	0.00	0.0351	0	0.00	0.0113
	II	37	1	0.18		1	0.15		0	0.07		0	0.08	
	III	22	2	0.25		1.5	0.19		0	0.11		0	0.11	
	IV	6	2.5	0.48		2	0.33		1	0.21		1	0.17	

## Discussion

Thus far, there is only one study reporting ADCP in ccRCC and the process was used to evaluate the effect of the monoclonal antibody against CAIX (35). Our report is, to date, the first study comprehensively reporting the cancer-intrinsic ADCP-regulatory gene signature in ccRCC. Although the intercellular process of phagocytosis has been elucidated, the cancer-intrinsic genetic alteration that drives or expels ADCP remains largely undecided, until a recent study by Kamber et al. (8). They employed an unbiased CRISPR/Cas9 screen and identified a reservoir of novel cancer-intrinsic genes involved in ADCP. We took advantage of the gene set and further generated a six-gene signature specific in ccRCC which showed an association not only with clinicopathological parameters but also with response to ICI and TKI.

Our study identified six genes that are associated both with ADCP and with prognosis in ccRCC. ARPC3 showed the highest HR and has not been reported in ccRCC. The gene encodes one of seven subunits of the human Arp2/3 protein complex (36). The Arp2/3 protein complex has been conserved through evolution and is implicated in the control of actin polymerization in cells (37). The process of phagocytosis is highly complex and involves major rearrangements of the cytoskeleton, a process in which the Arp2/3 protein complex plays a role (38). Padilla et al. reported that mir-124-5p regulates phagocytosis of human macrophages by targeting the actin cytoskeleton *via* the Arp2/3 complex (39). Silencing of the ARP2/3 complex has also been shown to disturb pancreatic cancer cell migration (40). Taken together, ARPC3 could be of both prognostic and therapeutical value in ccRCC, which warrants further study.

In contrast, PHF19 is not reported in phagocytosis but has vastly been reported in cancer research, most prominently in malignant melanoma (41). PHF19 is the subunit of polycomb repressive complex 2 (PRC2) and regulates the expression of key genes involved in cell growth and differentiation by modulating both PRC2/EZH2 catalytic activity and recruitment (42). Of note, its role has not been reported in ccRCC either. FKBP11 belongs to the FK506-binding protein (FKBP) that can bind specifically to the immunosuppressant FK506 and rapamycin (43). Whereas FKBP11 has not been reported in phagocytosis, immunosuppression of FK506 is vastly applied clinically in organ transplantation (44). Notably, an *in-silico* study using datasets overlapping ours also showed a similar prognostic role of FKFB11 in ccRCC (45). Neither MS4A14 nor KDELR3 was reported related to phagocytosis, and the genes are functionally distant from ADCP in previous studies. Identification of such genes shed light on further understanding of phagocytosis in ccRCC. Interestingly, CD1C is the only protective gene shown in our signature. Prior studies of CD1C in ccRCC focused on its expression in TILs and in peripheral lymphocytes showing increased CD1C-positive cells upon treatment of sunitinib and bevacizumab (46, 47). In our cohort, TIMER (https://cistrome.shinyapps.io/timer/) was used to determine the distribution of CD1C in ccRCC and found that it was predominantly expressed in tumor cells in ccRCC (48, 49). Our finding that CD1C expression was higher in the low-risk group that corresponded to lower IC50 of sunitinib echoes the previous studies, supporting the signature as a therapeutic marker.

Our findings could have translational implications. Besides prognosis, our signature was also associated with drug sensitivity. The sensitivity profile to TKIs and ICI corresponds to the current guidelines. We speculate that the low-risk group may benefit more from sunitinib and pazopanib whereas the high-risk group may benefit from ICI. Of note, TKIs that fall into later lines of treatment in ccRCC show inversed sensitivity between high- and low-risk groups from sunitinib and pazopanib, further echoing the fact that axitinib, tivozanib, sorafenib, and temsirolimus cannot reach first-line therapy, possibly due to the inherent ADCP status of patients. Interestingly, the high-risk group that showed lower IC50 in axitinib and sensitivity to ICI further corroborates the recent trial supporting the front-line use of axitinib-based ICI+TKI combination (50, 51). This also implies that the combination of sunitinib or pazopanib with ICI may not be as synergistic. Last but not least, we have listed several agents that could modify the ADCP status based on our signature, which may direct further investigation.

To find the optimal biomarker to predict prognosis and drug response in renal cell carcinoma, many gene signatures have been constructed based on the same methods in recent years. Wu et al. (52), Yin et al. (53), and Zhang Z et al. (54) constructed gene signatures according to differential expression genes between tumor and normal tissues; Zhang et al. (55) and Lv et al. (56) established gene signatures based on tumor microenvironment-related genes and glycolysis-related genes, respectively. They investigated the relationship between gene signatures and clinical features, immune infiltration, and prognosis, and these gene signatures had a good performance on predicting prognosis. Notably, the gene signature from Yin et al. demonstrated a significant correlation with response to immune checkpoint inhibitors (ICIs); the gene signature from Lv et al. was significantly associated with response to ICIs and tyrosine kinase inhibitors (TKIs), which might be the potential biomarker to predict drug response. Compared with previous similar studies, our research focused on ADCP-related genes and emphasized the important effect of ADCP-related genes on prognosis, tumor microenvironment, response to immunotherapy, and targeted therapy in ccRCC. Our monogram, integrating our gene signature and clinical features, possessed higher accuracy of prediction, whose AUC at 1 year reached 0.911. Additionally, we not only investigated the relationship between the gene signature and response to ICI but also predicted response to several common TKIs, which might contribute to developing a personalized treatment plan for patients with ccRCC. However, the patients enrolled in the validation cohorts were generally at the early stage and TKI usage was not enough to undergo statistical analyses.

Our study has limitations, First, this is an *in-silico* analysis without external validation. A complete elaboration of ADCP in ccRCC still requires profound *in vitro* and *in vivo* studies. Second, some of the drug-sensitivity analyses are profiled using simulation. A more rigorous drug screen is now in progress by our group. Finally, just like Kamber’s study, the ADCP-related genes were selected upon a CRISPR screen and many of its functionality in phagocytosis remains elusive. How the cancer-intrinsic signaling format ADCP by those genes should be thoroughly studied.

## Conclusion

Using an *in-silico* analysis by integrating multiple genomic datasets, we showed a six-gene ADCP signature that correlated with prognosis and immune modulation in ccRCC. The signature-based risk stratification was associated with response to both ICI and tyrosine kinase inhibition in ccRCC.

## Data Availability Statement

The datasets presented in this study can be found in online repositories. The names of the repository/repositories and accession number(s) can be found in the article/**Supplementary Material**.

## Ethics Statement

The studies involving human participants were reviewed and approved by the Huashan Institutional Review Board (HIRB). The patients/participants provided their written informed consent to participate in this study.

## Author Contributions

CF, YqL, XZ, and YfL carried out the *in silico* analysis. KL and CF participated in the study design. CF, HJ, and HW retrieved the data. CF and KL drafted the manuscript. All authors contributed to the article and approved the submitted version.

## Funding

This study was sponsored in part by the National Natural Science Foundation of China (Grant No. 81874123 and No. 81772709) and by the Beijing Advanced Innovation Center for Food Nutrition and Human Health, Beijing Technology and Business University (BTBU).

## Conflict of Interest

The authors declare that the research was conducted in the absence of any commercial or financial relationships that could be construed as a potential conflict of interest.

The reviewer FW declared a shared affiliation with the authors to the handling editor at the time of review.

## Publisher’s Note

All claims expressed in this article are solely those of the authors and do not necessarily represent those of their affiliated organizations, or those of the publisher, the editors and the reviewers. Any product that may be evaluated in this article, or claim that may be made by its manufacturer, is not guaranteed or endorsed by the publisher.
