# Corrigendum: Development of a phagocytosis- dependent gene signature to predict prognosis and response to checkpoint inhibition in clear-cell renal cell carcinoma

**DOI:** 10.3389/fimmu.2023.1140764

**Published:** 2023-01-19

**Authors:** Kunping Li, Yuqing Li, Yinfeng Lyu, Linyi Tan, Xinyi Zheng, Haowen Jiang, Hui Wen, Chenchen Feng

**Affiliations:** ^1^ Department of Urology, Huashan Hospital, Fudan University, Shanghai, China; ^2^ Department of Pharmacology, Huashan Hospital, Fudan University, Shanghai, China; ^3^ Beijing Advanced Innovation Center for Food Nutrition and Human Health, Beijing Technology and Business University (BTBU), Beijing, China

**Keywords:** clear-cell renal cell carcinoma, antibody-dependent cellular phagocytosis, immune checkpoint inhibition, biomarker, bioinformatics

In the published article, there was an error in the author contributions, and author Yinfeng Lyu should be added a token indicating equal contribution Yinfeng Lyu^†^. The corrected author list appears below.

Kunping Li ^1†^, Yuqing Li ^1†^, Yinfeng Lyu ^1†^, Linyi Tan ^1^, Xinyi Zheng ^2^, Haowen Jiang ^1*^, Hui Wen ^1*^ and Chenchen Feng ^1,2,3,*^

The authors apologize for this error and state that this does not change the scientific conclusions of the article in any way. The original article has been updated.

